# *E3L* and *F1L* Gene Functions Modulate the Protective Capacity of Modified Vaccinia Virus Ankara Immunization in Murine Model of Human Smallpox

**DOI:** 10.3390/v10010021

**Published:** 2018-01-04

**Authors:** Asisa Volz, Sylvia Jany, Astrid Freudenstein, Markus Lantermann, Holger Ludwig, Gerd Sutter

**Affiliations:** 1 Lehrstuhl für Virologie, Institut für Infektionsmedizin und Zoonosen, Ludwig-Maximilians-Universität München, 80539 Munich, Germany; asisa.volz@micro.vetmed.uni-muenchen.de (A.V.); sylvia.jany@micro.vetmed.uni-muenchen.de (S.J.); astrid.freudenstein@micro.vetmed.uni-muenchen.de (A.F.); 2 Deutsches Zentrum für Infektionsforschung (DZIF), 80539 Munich, Germany; 3 Division of Virology, Paul-Ehrlich-Institut, 63225 Langen, Germany; Markus.Lantermann@gmx.net (M.L.); Holger.Ludwig@cslbehring.com (H.L.)

**Keywords:** smallpox vaccination, MVA, immunogenic cell death, emergency vaccination

## Abstract

The highly attenuated Modified Vaccinia virus Ankara (MVA) lacks most of the known vaccinia virus (VACV) virulence and immune evasion genes. Today MVA can serve as a safety-tested next-generation smallpox vaccine. Yet, we still need to learn about regulatory gene functions preserved in the MVA genome, such as the apoptosis inhibitor genes *F1L* and *E3L*. Here, we tested MVA vaccine preparations on the basis of the deletion mutant viruses MVA-ΔF1L and MVA-ΔE3L for efficacy against ectromelia virus (ECTV) challenge infections in mice. In non-permissive human tissue culture the MVA deletion mutant viruses produced reduced levels of the VACV envelope antigen B5. Upon mousepox challenge at three weeks after vaccination, MVA-ΔF1L and MVA-ΔE3L exhibited reduced protective capacity in comparison to wildtype MVA. Surprisingly, however, all vaccines proved equally protective against a lethal ECTV infection at two days after vaccination. Accordingly, the deletion mutant MVA vaccines induced high levels of virus-specific CD8+ T cells previously shown to be essential for rapidly protective MVA vaccination. These results suggest that inactivation of the anti-apoptotic genes *F1L* or *E3L* modulates the protective capacity of MVA vaccination most likely through the induction of distinct orthopoxvirus specific immunity in the absence of these viral regulatory proteins.

## 1. Introduction

Variola virus (VARV), the causative agent of human smallpox, has been successfully eradicated by prophylactic vaccination using live vaccinia virus (VACV). However, even today more than thirty years after this famous achievement in medicine, there are still concerns that VARV may be used as bioterroristic weapon and zoonotic monkeypox or cowpox remain threatening infections in humans. Thus, developing improved vaccination principles ready to use in an immediate public health response are essential. The Modified Vaccinia virus Ankara (MVA) is a replication-deficient and safety tested VACV that is already licensed as a next-generation smallpox vaccine in Europe and Canada. Moreover, MVA has been actively investigated as a non-replicating multipurpose viral vector vaccine against other infections and cancer diseases [1,2]. Thus, MVA is a promising platform to develop innovative candidate vaccines inducing improved innate and adaptive immune responses. Scenarios of emergency immunization necessitate rapidly protective vaccination strategies. At best, this includes robust activation of immunity by preferably low dose applications of the respective vaccine. In previous studies, we and others confirmed the short-term protective capacity of low-dose MVA immunization in animal models for lethal infections with orthopoxviruses. Probably the best animal model for studying the protective capacity of new candidate orthopoxvirus-specific vaccines is the ectromelia virus-mouse challenge model. Ectromelia virus (ECTV), the causative agent of mousepox, provides an excellent surrogate model for human smallpox because a deadly systemic disease develops after inoculations of very low doses of virus [3]. Mice that survive the acute phase of infection develop a pustular rash on the skin, highly comparable to VARV infections in humans. Using the ECTV-mouse model, we confirmed that MVA vaccination efficiently prevents disease and death in C57BL/6 mice after lethal intranasal challenge infection [3,4,5]. However, with regard to the efficacy of immunization we still know rather little about the influence of regulatory gene functions preserved in the MVA genome. Indeed, inactivation of several MVA regulatory gene functions has already been confirmed as a strategy to further enhance immunogenicity and vaccine induced protective capacity [6,7]. More recently, viral proteins known to counteract induction of apoptosis [8,9,10,11] are amongst the most promising candidate targets for potential optimization of MVA as a vaccine. Programmed cell death as a trigger for enhanced immunogenicity can be explained by different mechanisms. Well-established is the hypothesis that apoptotic cells will be processed by dendritic cells resulting in an increased major histocompatibility complex (MHC) class I presentation for enhanced immunogenicity. In previous studies this so-called cross presentation has been proposed as the most important mechanism for antigen presentation upon primary immunization to induce efficient T-cell response [12]. In the context of cancer therapy, recent studies support the concept of the so called immunogenic cell death (ICD). ICD is another form of cell death that is reported to efficiently trigger an immune response against antigens associated with the dying cell. ICD has been initially identified when studying immune defense mechanisms against cancer cells. Here, the immunogenic death of cancer cells efficiently activated an effective antitumor immune response through antigen presentation by dendritic cells (DCs) and, consequently, activation of tumor-specific T cells. It has been hypothesized, that also virus infected cells might respond via this immunogenic form of apoptosis to counteract an infection. Of note, different from the tolerogenic, physiological cell death, ICD also triggers the activation of innate immunity including a wide range of proinflammatory cytokines and binding to pattern recognition receptors (PRRs) such as Toll-like receptor (TLR) 2 and 4 [13]. This will then further allow for enhanced activation of the adaptive immune responses [14,15]. Enhanced immunogenicity is reported for DNA vaccines with capacity to actively induce apoptosis [16,17], and improved antiviral immune responses are described for a dendritic cell vaccine against high grade glioma [13]. For VACV, several immune evasion proteins involved in inhibition of apoptosis/cell death have been identified. Of these, the open reading frames (ORF) *E3L* and *F1L* are functionally retained in the MVA genome [18,19,20,21,22]. VACV F1 protein acts in inhibiting the intrinsic pathway of apoptosis by localizing to the outer mitochondria membrane and repressing cytochrome C release. F1 targets the BH3-only protein Bim and prevents the activation of pro-apoptotic Bcl-2 family members Bak and Bax [11,23]. Once activated, these proteins play an important role for the activation of specific signal transduction pathways in the mitochondrial apoptotic effector machinery, resulting in the release of pro-apoptotic mediators like cytochrome c and subsequently in cell death [24]. The *F1L* gene encoding sequences are highly conserved among orthopoxviruses. In previous studies, deletion of the *F1L* gene in the VACV or MVA genome has been confirmed to enhance the induction of apoptosis after in vitro infection using different cell lines [19,23]. The VACV *E3L* gene encodes for a 25 kDa protein that features an amino-terminal Z-DNA-binding domain and a carboxyl-terminal RNA-binding domain. The carboxyterminal domain has been shown to inhibit double-stranded RNA (dsRNA)-activated protein kinase (PKR) by binding and sequestering dsRNA produced during VACV life cycle. Another function of the E3 polypeptide is to block phosphorylation and thus activation of interferon (IFN) regulatory factor 3 (IRF3) and IRF7, required for viral induction of IFN-α/β. In previous studies, an MVA *E3L* deletion mutant (MVA-ΔE3L) was found replication-deficient in chicken embryo fibroblasts (CEF) resulting in insufficient viral DNA and protein synthesis, enhanced induction of apoptosis, and increased production of chicken IFN-α/β [20]. The advantageous immunomodulatory properties of MVA vaccines are most likely based on the inactivation of VACV immune evasion genes that counteract the host immune response [2]. Thus, enhancing a potential MVA-mediated immunogenic cell death by inactivation of the *F1L* or *E3L* gene functions could be a promising approach to further improve the efficacy of MVA vaccination. However, the anti-apoptotic viral genes *F1L* and *E3L* are functional in MVA genome and the deletion of these genes also impairs the synthesis of late viral antigen in human and murine cells [20,21,22], and, therefore, their inactivation might lessen the efficacy of vaccination. To address this question, we generated and quality controlled vaccine preparations on the basis of the deletion mutant viruses MVA-ΔF1L and MVA-ΔE3L. As expected, the MVA deletion mutant vaccine viruses produced reduced levels of the VACV envelope antigen B5 upon tissue culture infection. When testing the candidate vaccines in the C57BL/6 mouse ECTV challenge model, the MVA vaccines lacking *E3L* or *F1L* genes exhibited reduced protective capacity in comparison to the MVA control vaccine at three weeks after single shot vaccination. Surprisingly, however, we found all vaccines equally efficient for rapidly protective immunization against a lethal ECTV challenge infection given two days after vaccination. In accordance with this finding, the deletion mutant MVA vaccines induced high levels virus-specific CD8+ T cells previously shown to be essential for rapidly protective MVA vaccination. These results suggest that inactivation of the anti-apoptotic genes *F1L* or *E3L* modulates the protective capacity of MVA vaccination against ECTV infection most likely through the induction of distinct orthopoxvirus specific immunity in the absence of these viral regulatory proteins.

## 2. Materials and Methods

### 2.1. Cells and Viruses

MA-104 (ATCC CCL-26), HeLa (ATCC CCL-2), NIH/3T3 (ATCC CRL-1658), BHK-21 (ATCC CCL-10) and chicken embryo fibroblast (CEF) cells were used and routinely maintained as previously described. Plaque purified Ectromelia virus (ECTV) strain Moscow (ATCC VR-1374, kindly provided by Mark L. Buller, St. Louis University School of Medicine, St. Louis, MI, USA) was propagated on MA-104 cells. As experimental vaccine served Modified Vaccinia virus Ankara (MVA) (clonal isolate F6; MVA F6) [25]; all recombinant, mutant and revertant viruses were derived from MVA F6. MVA-ΔE3L, MVA-ΔF1L and the corresponding revertant viruses were generated and characterized as described previously [19,20,21,22]. Viruses were routinely propagated and titrated on CEF or BHK-21 cells. Viral titers were determined by plaque assay and titrated in plaque forming units (pfu) as previously described [26].

### 2.2. Western Blot Analysis

Confluent monolayers of HeLa cells or NIH/3T3 cells were infected at a multiplicity of infection (MOI) of 5 with Modified Vaccinia virus Ankara (MVA) (clonal isolate F6), MVA-ΔF1L, MVA-ΔE3L and revertant viruses. Cell lysates were prepared at different time points after infection (3, 6, 9, 15 h post-infection (hpi). Lysates from uninfected cells or wild-type MVA-infected cells served as controls. Polypeptides were separated by sodium dodecyl sulfate polyacrylamide gel electrophoresis (SDS-PAGE) and electroblotted onto a polyvinylidene difluoride (PVDF) membrane. After blocking, membranes were incubated with primary antibodies (rabbit anti-B5 diluted 1:500; rabbit anti-C7 diluted 1:1000; rabbit anti-poly(ADP-ribose)polymerase (PARP) Cell Signaling Technology (Danvers, MA, USA) diluted 1:1000; mouse anti-β-Actin Sigma-Aldrich (St. Louis, MI, USA) diluted 1:1000) at 4 °C overnight. After washing, the blots were incubated with secondary antibodies for one hour at room temperature.

### 2.3. Northern Blot Analysis

For analysis of mRNA, cells were infected with MVA, MVA-ΔE3L or the revertant virus MVA-ΔE3Lrev at an MOI of 5. PBS-infected cells served as mock-control. Total RNA was isolated with TRIzol reagent (Invitrogen, Carlsbad, CA, USA), following the manufacturer’s instructions. Total RNA was separated by electrophoresis in 1% agarose formaldehyde gels. Following, RNA was transferred onto positively charged nylon membranes (Roche Diagnostics, Basel, Switzerland). Riboprobes for detection of MVA-encoded *047R* (*F17R*) mRNAs and reverse primers containing a T7-RNA polymerase promoter were synthesized as previously described [21]. In vitro RNA labeling, hybridization, and signal detection were carried out according to the manufacturer’s instructions (Digoxigenin-UTP (DIG) RNA labeling kit and detection chemicals; Roche Diagnostics, Basel, Switzerland), applying 68 °C for prehybridization, hybridization, and high stringency washing.

### 2.4. Mice

Female C57BL/6N mice (6–10 weeks old) were purchased from Charles River Laboratories (Sulzfeld, Germany). For experimental work, mice were housed in an isolated (ISO) cage unit (Tecniplast, Hohenpeißenberg, Germany) and had free access to food and water. All animal experiments were handled in compliance with the German regulations for animal experimentation (Animal Welfare Act, approved by the Government of Upper Bavaria, Munich, Germany).

### 2.5. Immunization and Infection Experiments

Intramuscular (i.m.) vaccination was performed by injection of 50 µL of virus suspension containing 10^5^ or 10^6^ pfu of MVA, MVA-ΔF1L, MVA-ΔE3L or phosphate-buffered saline (PBS) into the left hind leg. For intranasal infection, mice were anesthesized by intraperitoneal (i.p.) injection with 1 mg ketamine and 0.04 mg xylazine per 10 g body weight. Intranasal infection was performed by instillation of 20 µL of virus suspension containing 200 pfu (~3 × LD_50_) or 2000 pfu (~30 × LD_50_) ECTV. Signs of illness, weight loss and survival were monitored daily for at least three weeks. In all experiments inoculations of corresponding amounts of PBS were used as controls (mock vaccine).

### 2.6. Analysis of MVA-Specific Antibodies

Firstly, 96-well immunoplates (Nunc-Immuno^TM^ MicroWell^TM^ 96 well solid plates, Sigma-Aldrich, Munich, Germany) were coated with 2 µg/mL sucrose gradient-purified MVA F6 in coating buffer (70 mM NaHCO_3_, 30 mM Na_2_CO_3_, pH 9.6) for 3 h at 37 °C and overnight at 4 °C. After blocking with PBS supplemented with 10% heat-inactivated fetal calf serum and 0.05% Tween-20, the plates were incubated with serial dilutions of sera from immunized mice. Antibody titers were determined using an alkaline-phosphatase conjugated anti-mouse antibody and p-nitrophenyl phosphate (Sigma-Aldrich, Munich, Germany) as substrate.

### 2.7. Analysis of MVA Neutralizing Antibodies

For analysis of MVA neutralizing antibodies we used the reference clonal isolate MVA F6 purified by ultracentrifugation through sucrose cushions. Serum samples from immunized animals or mock-vaccinated animals were tested in duplicate or triplicate in plaque reduction assays on CEF cell monolayers grown in 24-well tissue culture plates. Eight two-fold serial dilutions of serum were mixed with 200 pfu of MVA F6 at 37 °C for 2 h. Following incubation, 100 μL of the reaction mix was loaded onto the CEF cell monolayers. Cells were over-laid with medium and incubated at 37 °C with 5% CO_2_ for 48 h. In the following media were removed and the cells were fixed with ice-cold methanol-aceton for 5 min. Fixed cell monolayers were immunostained using primary rabbit anti-VACV antibody (diluted 1:2000) and secondary antibody (peroxidase labeled goat anti-rabbit-Ig, Dianova, Hamburg, Germany) followed by incubation with True Blue Peroxidase Reagent (KPL, medac GmbH, Wedel, Germany). Blue plaques were counted and MVA neutralization titers were determined to be the last dilution of serum that reduced the number of plaques by 60% compared with control wells.

### 2.8. Analysis of Antigen-Specific CD8+ T Cells by Enzyme-Linked Immunospot Assay (ELISPOT)

Mice were sacrificed 8 or 56 days post-immunization. A cell suspension was prepared by homogenizing the spleens through 200-µm mesh sieves, and red blood cells were removed by adding red cell lysis buffer (Sigma, St. Louis, MI, USA). After centrifugation, the cell pellet was dissolved in Roswell Park Memorial Institute (RPMI) medium supplemented with 10% fetal calf serum, 2 mM l-glutamine and 100 IU/mL penicillin-streptomycin. Interferon gammas (IFN-γ)-secreting CD8+ T cells were analyzed by using the Enzyme Linked Immuno Spot Assay (ELISPOT) PLUS kit for mouse IFN-γ (MABTECH, SE-131 28 Nacka Strand, Sweden) following the manufacturer’s instructions. ELISPOT plates were preincubated overnight with the antibody solution and then incubated with the cell suspension that had been stimulated with the virus-specific peptide B8R_20–27_ (TSYKFESV, [27]). The spots were counted and analyzed by using an automated ELISPOT plate reader and software following the manufacturer’s instructions (A.EL.VIS Eli.Scan software; A.EL.VIS, Hanover, Germany).

### 2.9. Statistical Analysis

Statistical comparison of different groups of mice was analyzed by one-factorial analysis of variance (ANOVA) for the area under the percentage-of-initial-weight curves (AUC). The differences between vaccination groups were analyzed with a one-factorial analysis of variance model. For multiple comparisons, *P* values were adjusted with the Bonferroni method. CD8+ T cell responses were compared by T test. The statistical evaluation was performed with GraphPad Prism for Windows (GraphPad Prism Software, La Jolla, CA, USA).

## 3. Results

### 3.1. Decreased Synthesis of the Late Viral Protein B5 in the Absence of E3L or F1L Gene Expression

**Figure 1:**
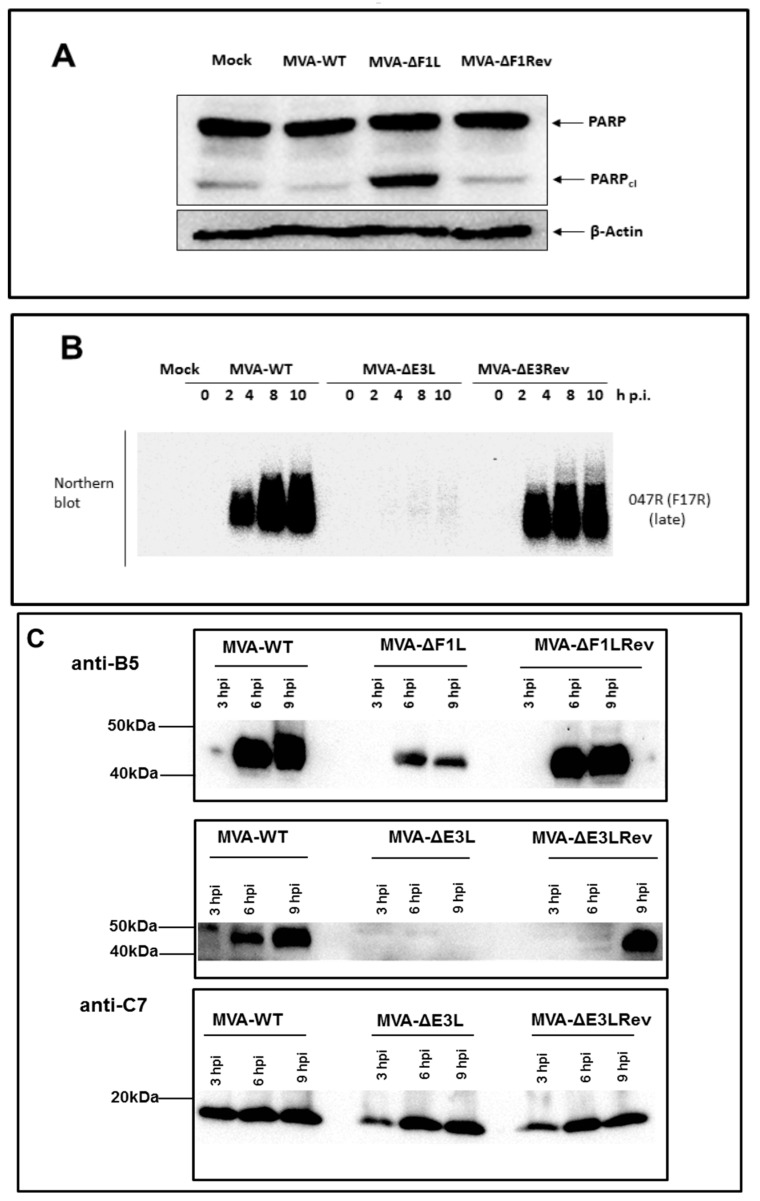
(**A**) Western blot analysis of deletion mutant virus MVA-ΔF1L-induced apoptosis in murine cells. Lysates of NIH/3T3 cells infected with Modified Vaccinia virus Ankara (MVA), MVA-ΔF1L or MVA-ΔF1Lrev at a multiplicity of infection (MOI) of 5 were prepared 15 hpi, proteins were separated by sodium dodecyl sulfate polyacrylamide gel electrophoresis (SDS-PAGE) and immunoblotted against poly-ADP ribose polymerase (PARP)/PARPcl and β-Actin as loading control. (**B**) Inhibition of late gene expression in MVA-ΔE3L infected HeLa cells. Total RNA was isolated and viral late mRNA was monitored by Northern blotting, using riboprobes specific for MVA late gene *047R*. (**C**) Western blot analysis of MVA proteins produced in HeLa cells. Lysates of cells infected with deletion mutant viruses MVA-ΔF1L and MVA-ΔE3L were prepared at three, six, or nine hpi. Polypeptides were separated by SDS–PAGE and tested by immunoblotting using specific polyclonal antibodies against vaccinia virus (VACV) B5 protein and VACV C7 protein. Lysates from uninfected cells (Mock) or wildtype MVA infected cells (MVA-WT) or revertant viruses (MVA-ΔF1Lrev, MVA-ΔE3Lrev) infected cells served as controls. Indicated on the left are the positions of the molecular weight markers in comparison to the ~45 kDa VACV B5 (B5) and ~17 kDa VACV C7 (C7) proteins.

Activation of the MVA life cycle in most host cells results in the synthesis of all classes (early, intermediate, and late) of VACV proteins. Among the viral early proteins are antigens containing important determinants of T cell immunity that are presented via MHC-I molecules to CD8+ T cells [27,28]. Late viral proteins include surface antigens that are key targets of VACV neutralizing antibodies such as B5 a major surface protein of extracellular VACV virions [29]. Thus, we monitored the production of the viral protein B5 in HeLa cells infected with MVA, MVA-ΔF1L or MVA-ΔE3L by Western blot analysis. The revertant viruses MVA-ΔF1Lrev and MVA-ΔE3Lrev served as additional controls. As shown by the monitoring for cleaved poly-ADP ribose polymerase (PARP), the characteristic induction of apoptosis in cells infected with MVA-ΔF1L is reversed upon infection with the revertant virus (Figure 1A). Similarly, the revertant virus MVA-ΔE3Lrev overturned the failure of MVA-ΔE3L to proceed to late gene expression upon the infection of HeLa cells as demonstrated by the Northern blot detection of significant amounts of late mRNA specific for the MVA gene *047R* (*F17R*) (Figure 1B). Previous work had already demonstrated that MVA-ΔE3L is deficient in late protein production in murine and human cells [21,22]. Thus, as expected, we easily found B5 in the lysates of cells infected with MVA or the revertant viruses at six and nine hpi, and, we failed to detect B5 in the lysates of MVA-ΔE3L infected cells (Figure 1C). Though our Western blot was at best a semi-quantitative assay, the synthesis of B5 protein in MVA-ΔF1L infected cells was apparent at six and nine hpi; however, the amounts of B5 revealed with MVA-ΔF1L were clearly reduced compared to those detected with MVA or the revertant virus MVA-ΔF1Lrev. To control for uniform infections with MVA-ΔE3L or the control viruses, and to confirm the unimpaired activation of viral early gene expression, we also monitored for the synthesis of the early viral protein C7. At three, six, nine hpi, as expected, we detected comparable levels of C7 protein in the lysates of HeLa cells infected with MVA, MVA-ΔE3L and MVA-ΔE3Lrev. These results indicated that an inactivation of either the *F1L* or the *E3L* gene function does interfere with the synthesis of late but not early gene products in MVA vaccine infected mammalian cells.

### 3.2. Protective Capacity of MVA Deletion Viruses against Lethal Poxvirus Infections

**Figure 2:**
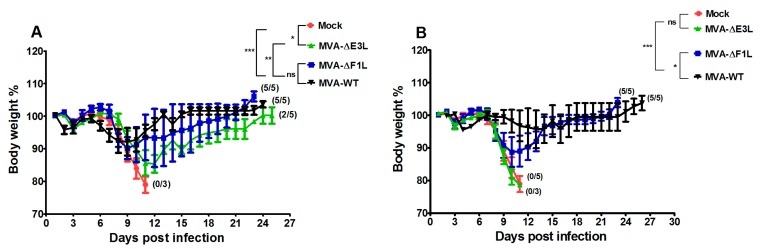
Protective capacity of MVA vaccination against lethal mousepox virus infections. C57BL/6 mice were challenged with 2000 plaque forming units (pfu) ECTV 21 days after immunization with MVA vaccine at a dose of 10^6^ pfu (**A**) or a dose of 10^5^ pfu (**B**) or PBS (mock vaccinated animals, used as controls). In all experiments, weight loss of individual mice was monitored daily (three to five per group). Error bars indicate standard errors of the means (SEMs), and the numbers of surviving/total animals are given in parentheses. * *p* < 0.05; ** *p* < 0.01; *** *p* < 0.001. Data are representative of two or three experiments.

To elucidate the role of the F1 and E3 proteins in protective immunization, we tested MVA-ΔF1L and MVA-ΔE3L in the C57BL/6 mouse-ECTV challenge model [3,4,5,30]. Briefly, we intramuscularly (i.m.) vaccinated mice with either 10^5^ or 10^6^ pfu MVA-ΔF1L and MVA-ΔE3L twenty-one days before the high dose respiratory infection with ECTV (2000 pfu corresponding to about 30 × LD_50_) (Figure 2). Control mice were mock vaccinated with PBS. In the groups receiving 10^6^ pfu of MVA or MVA-ΔF1L vaccine all mice survived the harsh ECTV challenge infection. Interestingly, we observed a clearly reduced protective capacity of MVA-ΔE3L vaccination with only two out of five mice surviving the challenge. Furthermore, we noticed differences among the groups when monitoring for signs of disease following the ECTV infection. MVA vaccination induced robust protection against the challenge without any signs of illness or detectable body weight loss. Immunization with MVA-ΔF1L prevented death of the mice. However, all vaccinated animals developed signs of mousepox disease and suffered from about 10% body weight loss starting six days post infection (dpi). In MVA-ΔE3L vaccinated mice, the signs of disease were more severe and culminated with a loss of up to 15% of the body weight at day eleven post infection. Inoculations with doses of 10^5^ pfu of the MVA vaccines resulted in a more pronounced but similar outcome with regard to the induction of protective immunity against the high-dose ECTV infection. All mice that had been vaccinated with 10^5^ pfu MVA-ΔE3L developed systemic mousepox disease with progressive body weight loss starting nine dpi, and died or had to be euthanized until thirteen dpi. In contrast, vaccinations with 10^5^ pfu of either the MVA-ΔF1L or MVA vaccine protected against lethal mousepox. MVA-ΔF1L vaccinated mice, however, developed severe signs of illness which correlated with the body weight loss. In contrast, in MVA immunized mice we observed no obvious weight loss or signs of mousepox illness. Taken together, our data indicate that in comparison to standard MVA, vaccination immunizations with the MVA mutant viruses deleted in the regulatory genes F1L or E3L have a reduced capacity to induce protective immunity against severe mousepox.

### 3.3. MVA-ΔF1L or MVA-ΔE3L Vaccines Prime Unimpaired VACV-Specific CD8+ T-Cell Responses

**Figure 3:**
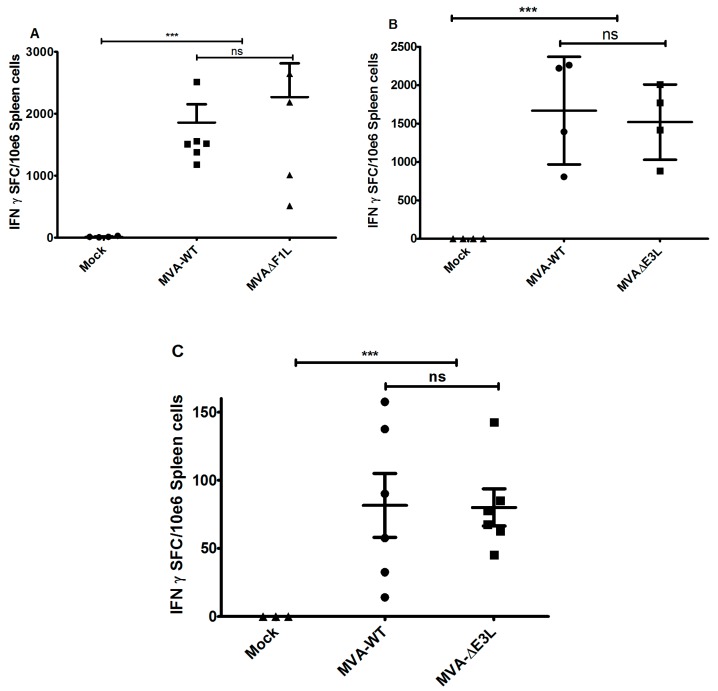
Virus-specific CD8+ T cells elicited by MVA vaccination. C57BL/6 mice were inoculated intramuscularly with 10^6^ pfu MVA, MVA-ΔF1L (**A**), MVA-ΔE3L (**B**), or PBS. At eight days post-vaccination, splenocytes were prepared, and B8R_20–27_-specific IFN-γ-producing CD8+ T cells were measured by Enzyme Linked Immuno Spot Assay (ELISPOT). Virus-specific CD8+ T cell memory responses elicited by MVA-WT, MVA-ΔE3L vaccination or PBS in C57BL/6 mice (*n* = 3–6 per group) (**C**). Splenocytes were prepared at 56 days after prime vaccination. B8R_20–27_–epitope-specific, IFN-γ spot forming CD8+ T cells (IFN-γ SFC) were quantified by ELISPOT. Data are representative of two similar experiments. *** *p* < 0.001; ns, not significant.

In previous work, we showed that vaccine induced virus specific CD8+ T cell responses are essential for rapidly protective vaccination with MVA or conventional vaccinia virus in the ECTV mouse challenge model [3]. Our data so far suggested a restricted capacity of MVA-ΔE3L and MVA-ΔF1L vaccination to confer protection against lethal mousepox twenty-one days after vaccination. To further assess the candidate vaccine induced T cell responses we vaccinated C57BL/6 mice via the intramuscular route using 10^6^ pfu of MVA, MVA-ΔF1L or MVA-ΔE3L and analysed VACV-specific CD8+ T cells by IFN-γ-ELISPOT. Single intramuscular immunizations with all test vaccines induced substantial levels of B8R_20–27_-epitope-specific CD8+ T cells with median absolute numbers of 3.15 × 10^3^ IFN-γ spot forming cells (SFC) per 10^6^ splenocytes. Of note, MVA, MVA-ΔF1L and MVA-ΔE3L vaccines induced close to identical magnitudes of CD8+ T cell responses (Figure 3A,B). These data suggest an unimpaired priming of orthopoxvirus-specific CD8+ T cells following immunization with MVA-ΔF1L or MVA-ΔE3L. Of note, MVA and MVA-ΔE3L vaccines elicited very comparable levels of B8R_20–27_-epitope-specific memory CD8+ T cells as demonstrated by the IFN-γ-ELISPOT analysis of T cell responses at 56 days after initial immunization (Figure 3C).

### 3.4. Rapidly Protective Immunization with MVA Vaccines Deficient in E3L or F1L

**Figure 4:**
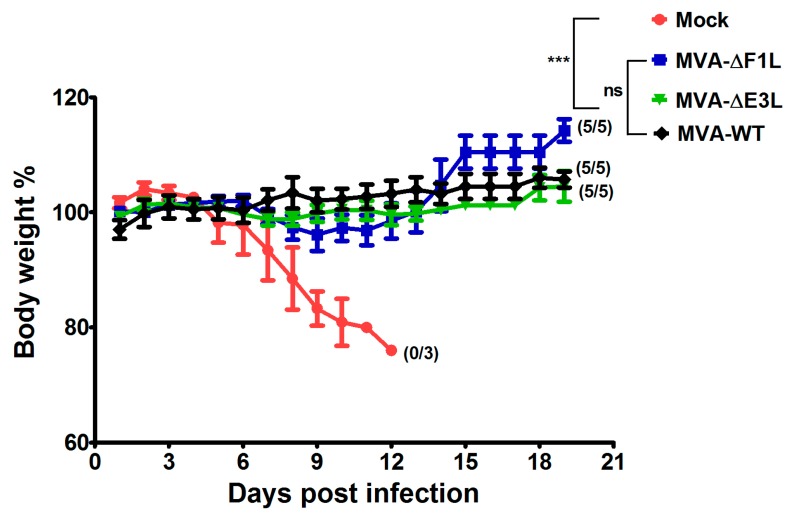
Short-term protective capacity of MVA vaccination against lethal mousepox virus challenge infection. Groups of C57BL/6 mice (*n* = 5) were inoculated with 200 pfu ECTV two days after immunization with 10^5^ pfu of MVA, MVA-ΔF1L, or MVA-ΔE3L. PBS vaccinated animals (*n* = 3) served as controls (Mock). In all experiments, weight loss of individual mice was monitored daily. Error bars indicate standard errors of the means (SEMs), and the numbers of surviving/total animals are given in parentheses. *** *p* < 0.001. Data are representative of two or three experiments.

A sought-after feature of next-generation smallpox vaccines is the capacity to elicit rapidly protective immunity against severe orthopoxvirus infections. In previous work, we demonstrated that even a low dose of MVA vaccine (10^5^ pfu) is sufficient to robustly protect against a lethal dose of ECTV inoculated only two days after immunization [3]. Thus, we also evaluated the MVA-ΔF1L and MVA-ΔE3L candidate vaccines in this model for short-term protection by vaccination. C57BL/6 mice received intramuscularly 10^5^ pfu of either MVA, MVA-ΔF1L, or MVA-ΔE3L vaccine. Injections with PBS served as mock vaccines. Two days after immunization, mice were intranasally inoculated with ECTV (200 pfu corresponding to approximately 3 × LD_50_) [3,4]. As expected, mock-vaccinated control mice developed systemic mousepox disease with progressive body weight loss and died within thirteen days after challenge. In accordance with previous data, all mice vaccinated with MVA mounted high level protective immunity to the ECTV challenge infection. Remarkably, also immunizations with the MVA-ΔE3L or MVA-ΔF1L candidate vaccines fully protected against mousepox disease and death without observation of any signs of illness or body weight-loss. These data clearly suggested that the lack of the VACV proteins F1 and E3 did not influence the outcome of rapid protection against a lethal systemic orthopoxvirus infection in the context of MVA vaccination (Figure 4).

## 4. Discussion

The replication-deficient and safety tested virus MVA has been licensed as a third-generation smallpox vaccine in Europe and in Canada to replace the conventional live vaccinia virus vaccines [31]. The purpose of this study was to evaluate the possibility for further improvement of MVA as smallpox vaccine including the development of vaccines that confer rapid protection in a case of emergency. Here, we used the ECTV mousepox model to test the protective capacity of MVA vaccines lacking the ability to produce the VACV regulatory proteins E3 or F1. These are among the most potent VACV regulatory immune defense proteins being produced by MVA and target the activation of host innate immune responses and the onset of apoptosis [2]. The *E3L* and *F1L* gene sequences are fully conserved in the MVA genome and the deletion mutant viruses MVA-ΔF1L and MVA-ΔE3L demonstrate impaired viral life cycles and elicit increased levels of cell death in various cell types [19,20]. In particular, the expression of late viral genes is compromised and reduced amounts of MVA late structural proteins are made upon infections with the mutant viruses. This phenotype is associated with the inhibition of viral intermediate transcription in MVA-ΔE3L infected HeLa cells [21,22]. Hereby, the lack of the regulatory protein E3 results in an increased type I interferon response, the induction of apoptosis, and an enhanced synthesis of other cytokines and chemokines [19,20,32,33]. These findings are in line with a major function of VACV E3 to bind and sequester double-stranded RNA (dsRNA), thereby preventing the activation of type I interferons and important interferon response proteins such as PKR and 2′-5′ oligoadenylate synthetase (OAS) [34,35]. Correspondingly, a similar enhancement of innate immune responses is observed with recombinant MVA engineered to express excess amounts dsRNA early during infection and in the presence of a functional E3L gene [36]. Inevitably, the impaired late gene expression in MVA-ΔE3L infections was likely to result in reduced synthesis of MVA structural antigens. Indeed, when monitoring *in vitro* antigen production by our MVA-ΔF1L and MVA-ΔE3L candidate vaccines we clearly demonstrate a decreased synthesis of the VACV envelope antigen B5 in human HeLa cells (Figure 1C). As B5 is a relevant target protein for virus-neutralizing antibodies in humans these results questioned the suitability of *E3L* and *F1L* gene inactivations to increase MVA immunogenicity as a smallpox vaccine. Yet, on the other hand the inactivation of the *F1L* gene in MVA is reported to enhance CD8+ T cell responses to the vector delivered HIV-1 target antigens [37]. In our study, the inhibition of B5 antigen production was clearly more pronounced in infections with MVA-ΔE3L compared to MVA-ΔF1L. This result is in agreement with the distinct interruption of the molecular virus life cycle in MVA-ΔE3L infected HeLa cells (Figure 1B). The finding of reduced B5 production in MVA-ΔF1L infection seems mostly the consequence of enhanced apoptosis limiting the overall time and resources for viral protein synthesis due to the absence of the anti-apoptotic protein F1. Thus, both deletion mutant viruses MVA-ΔE3L and MVA-ΔF1L were expected to deliver reduced levels of VACV late structural antigens but also to exhibit particular immunostimulatory features. In consequence, the ability of MVA-ΔE3L and MVA-ΔF1L to elicit protective immunity against orthopoxvirus infections was unclear. To comparatively test the protective capacity of the MVA vaccines we chose lethal infections of mice with ECTV, a learned animal model for human smallpox. Of note, we used two different models of respiratory ECTV infections: (i) at two days after vaccine inoculation a 200 pfu ECTV challenge (~3 × LD_50_) to test for rapidly protective immunization, and (ii) at 21 days after vaccination a 2000 pfu ECTV challenge (~30 × LD_50_) resulting in pronounced respiratory disease and early death of infected animals to evaluate the protective capacity of a more conventional orthopoxvirus-specific immunization. The vaccines were given by intramuscular inoculation (a route routinely used for immunization in humans) and at doses (10^5^ or 10^6^ pfu) that were known to be fully protective against mousepox virus challenge infection [3,5,30] but are about 100-fold reduced compared to the usual dosage of MVA vaccines in humans [38,39]. Our findings in the model for conventional immunizations suggested, that MVA-ΔE3L vaccination is significantly less protective compared to vaccinations with MVA-ΔF1L or MVA. This reduced protection observed with MVA-ΔE3L is likely best explained by the clearly impaired viral late gene expression resulting in minor amounts of structural antigens and an insufficient activation of virus-specific antibodies and T cells. In the model of the harsh 2000 pfu ECTV challenge infection vaccine induced antibody responses are likely needed for protection. Indeed, virus-specific antibodies have been shown to play a dominant role for protection against highly lethal orthopoxvirus challenge infections in other mouse and monkey models [40,41,42]. Thus, we suspected delivery of an insufficient amount of structural antigen as a possible explanation for the impaired protective efficacy of MVA-ΔE3L immunization. However, a standard comparison of antibody responses elicited in mice receiving 10^6^ pfu MVA, MVA-ΔF1L or MVA-ΔE3L vaccine did not reveal differences in the levels of virus particle binding or virus neutralizing antibodies (data not shown). Our failure seems to reflect the common inability of standard immune monitoring assays to reveal correlates for vaccine-mediated protection in vivo. Clearly, more detailed studies are needed to identify the distinct correlate(s) of protection induced by conventional MVA immunization in comparison to MVA-ΔE3L in our ECTV mouse challenge model. Orthopoxviruses induce complex antibody responses reactive against many diverse antigens [42,43]. Therefore, we might have missed the crucial antibody response specificities targeting critical antigens when using our standard immune monitoring assays based on the measurement of MVA-binding or MVA-neutralizing antibodies. Moreover, besides antibody responses the likely immunologic mechanisms underlying protection by VACV immunization are diverse including CD4+ and CD8+ T cells [44]. Since the essential relevance of cytotoxic CD8+ T cells for early control of primary ECTV replication in C57BL/6 mice is well documented for MVA vaccination [3,4], we hypothesized that the absence of anti-apoptotic F1 or E3 protein might inadvertently influence the activation of CD8+ T cell immune responses. Yet, all test vaccines allow for an equally efficient priming of B8R_20–27_ specific CD8+ T cells. This observation is in accordance with the results from other studies suggesting VACV early proteins as major source for virus-specific CD8+ T cell determinants in mice [27,28,45]. We previously showed that virus-specific CD8+ T cells serve as essential component for a rapidly protective immune response against ECTV challenge infection [3]. Indeed, when comparing the efficacy of MVA-ΔE3L, MVA-ΔF1L or MVA we revealed full protection with vaccines given just two days prior to the lethal ECTV infection. Of note, we can assume that the immune mechanism of rapid protection is different to that in conventional protective vaccination. The unimpaired efficacy of MVA-ΔE3L in rapidly protective vaccination is best explained by the fact that protection in this mousepox model is solely based on rapidly activated T cell immunity. In contrast, the conventional vaccination with MVA-ΔE3L cannot protect against a harsh lethal lung infection presumably due to the failure to elicit essential antibody responses in addition to T cell immunity. The mousepox challenge infection in a time range very close to the MVA immunization greatly enhances the development of antiviral immunity allowing for efficient ECTV clearance. This scenario seems to compensate for the apparently impaired ability of the MVA-ΔE3L vaccine to establish or maintain protective levels of immunity at twenty-one days post immunization. Currently, the immune correlate(s) for the deficiency of MVA-ΔE3L immunization are unclear. MVA-ΔE3L might fail to produce the target antigens for essential immune response or it induces insufficient virus-specific memory responses. Worth again mentioning is that the MVA infection in absence of the immune regulatory protein E3 will result in a potent activation of innate immune responses [32,33]. Thus, MVA-ΔE3L as a candidate vaccine might be further considered for CD8+ T cell priming and potentially for superior delivery of recombinant T cell antigens.

## 5. Conclusions

Our study in an experimental murine model for human smallpox suggests that MVA vaccines with inactivated regulatory genes *E3L* or *F1L* efficiently prime antigens-specific CD8+ T cells, allow for rapidly protective immunization against a lethal ECTV infection but do not improve the overall protective efficacy of MVA in conventional vaccination against a harsh respiratory ECTV challenge.
